# Barriers to Participation in a Patient Satisfaction Survey: Who Are We Missing?

**DOI:** 10.1371/journal.pone.0026852

**Published:** 2011-10-26

**Authors:** Angèle Gayet-Ageron, Thomas Agoritsas, Laura Schiesari, Véronique Kolly, Thomas V. Perneger

**Affiliations:** 1 Division of Clinical Epidemiology, Department of Community Health and Medicine, University of Geneva, University Hospitals of Geneva, Geneva, Switzerland; 2 Quality of Care Service, University of Geneva, University Hospitals of Geneva, Geneva, Switzerland; 3 Quality of Care Unit, Hospital Sirio-Libanès, Sao Paulo, Brazil; 4 Office of Mediation, University Hospitals of Geneva, Geneva, Switzerland; Yale University School of Medicine, United States of America

## Abstract

**Background:**

A common weakness of patient satisfaction surveys is a suboptimal participation rate. Some patients may be unable to participate, because of language barriers, physical limitations, or mental problems. As the role of these barriers is poorly understood, we aimed to identify patient characteristics that are associated with non-participation in a patient satisfaction survey.

**Methodology:**

At the University Hospitals of Geneva, Switzerland, a patient satisfaction survey is regularly conducted among all adult patients hospitalized for >24 hours on a one-month period in the departments of internal medicine, geriatrics, surgery, neurosciences, psychiatry, and gynaecology-obstetrics. In order to assess the factors associated with non-participation to the patient satisfaction survey, a case-control study was conducted among patients selected for the 2005 survey. Cases (non respondents, n = 195) and controls (respondents, n = 205) were randomly selected from the satisfaction survey, and information about potential barriers to participation was abstracted in a blinded fashion from the patients' medical and nursing charts.

**Principal Findings:**

Non-participation in the satisfaction survey was independently associated with the presence of a language barrier (odds ratio [OR] 4.53, 95% confidence interval [CI95%]: 2.14–9.59), substance abuse (OR 3.75, CI95%: 1.97–7.14), cognitive limitations (OR 3.72, CI95%: 1.64–8.42), a psychiatric diagnosis (OR 1.99, CI95%: 1.23–3.23) and a sight deficiency (OR 2.07, CI95%: 0.98–4.36). The odds ratio for non-participation increased gradually with the number of predictors.

**Conclusions:**

Five barriers to non-participation in a mail survey were identified. Gathering patient feedback through mailed surveys may lead to an under-representation of some patient subgroups.

## Introduction

Patient-reported outcomes, such as patient evaluations of care or self-reported functional status, are increasingly used as indicators of health care quality [1], [2]. These indicators are often assessed by means of mailed surveys. A common weakness of such surveys is a suboptimal participation rate [3], [4]. This raises the threat of selection bias [5]–[8], and calls in question the validity of the results.

**Figure 1:**
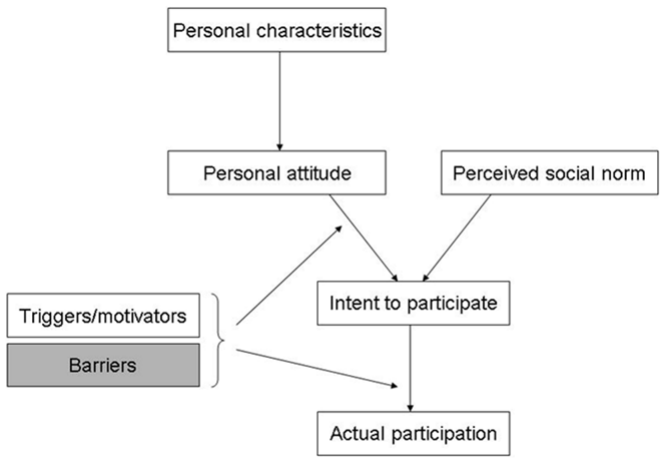
Mechanism of participation in a patient satisfaction survey. This study examined barriers to participation (grey box).

It is useful to break down the mechanism of participation in a survey in order to better understand it. A simple framework for the mechanisms of non-participation can be proposed (Figure 1). Actual participation is preceded by an intention to participate, which is influenced by a personal attitude toward the survey, and the perceived social norm regarding such surveys. Personal characteristics [9], [10], familiarity with surveys, and the relationship with the survey sponsor [11], [12] or the study personnel [13] will influence the personal attitude. In addition, other factors will influence the intent to participate and actual participation in a positive or a negative way. The intention to participate can be enhanced by the perceived importance of the topic, the lack of intrusiveness of the questions, a convincing cover letter, the assurance of confidentiality, evidence of a review by an ethics committee, and a variety of incentives, such as small gifts [14]–[17]. The act of filling out the questionnaire and sending it back will be facilitated by the reasonable length and appealing layout of the questionnaire [15], clearly worded questions and response options, and the provision of a prepaid mail-back envelope [14]. Finally, a person may have the intention of responding, yet may be unable to do so. This inability is due to an incompatibility between the survey methods and the intended respondent's abilities; we call such incompatibilities “barriers”. In a mailed survey, the following barriers may impede participation: language or cultural barriers [18], [19], illiteracy [20], difficulty in reading or writing due to sensory or motor deficiencies, and difficulty in understanding what is required due to cognitive limitations [21], [22], drug use [23], or mental illness.

Current knowledge about non-participation in health surveys [9], [21], [24], [25] concerns predominantly patient socio-demographic characteristics. Other variables that have been explored include patient satisfaction with care (in satisfaction surveys) [5], [26] or use of health services [6]. Nonetheless little is known about the importance of barriers to participation, i.e., barriers directly explaining non-participation. Knowledge of barriers is important because it can suggest ways of overcoming them, and barring that, it can help better understand the potential implications of the respondent self-selection process. We hypothesized that non-French speaking patients who had limited knowledge of French would be less likely to participate because of their inability to read the questionnaire or to complete the associated inviting letter. Other possible barriers might include a sight deficiency or difficulties in writing, a cognitive impairment due to a neuro-psychiatric disease or secondary to a substance or alcohol abuse. By studying a large population of patients with various medical problems and not by selecting patients on sex, age or on specific pathologies as it was frequently done [3], [10], [21], [23], [27], we aim to better explore these hypotheses.

In this study, we assessed barriers to participation in a patient satisfaction survey conducted at a large teaching hospital, using a case-control study design that compared participants to non-participants.

## Materials and Methods

### Ethics Statement

This case-control study was approved on September 25^th^, 2006 as a separate research project by the Research Ethics Committee of the University Hospitals of Geneva. In particular, examination of medical records was not part of the original patient survey design and required specific authorization. As the original survey guaranteed that questionnaire responses would not be linked with the participants' identification, satisfaction scores of the survey participants were not retrieved and analysed.

### Study setting

The study was conducted at the University hospitals of Geneva, a 1900-bed public teaching hospital located in Geneva, Switzerland. Activity represents >700'000 hospitalization days and >47'000 admissions per year.

### Patient satisfaction survey

Patient satisfaction surveys are conducted on a regular basis at the hospital [26]. The survey considered in this study was realized among adult patients hospitalized for more than 24 hours in the departments of internal medicine, geriatrics, surgery, neurosciences, psychiatry, and gynaecology-obstetrics and discharged between September 15 and October 15, 2005 to their usual place of residence (transfers to other hospitals were excluded). The patient list was obtained from the administrative office of the University hospitals of Geneva using the criteria on minimum hospital stay duration, home address availability, and vital status. Patients selected for the satisfaction survey received a questionnaire by mail on November 15, 4–8 weeks after discharge. The survey packet included a cover letter explaining the purpose of the study, indicating that participation was appreciated but voluntary and providing contact information, the questionnaire and a business-reply envelope. All survey materials were in French. Two reminders were sent 1 month and 2 months after the initial mailing. The initial list included 2469 patients, of whom 229 were further excluded because of death (n = 46), mail return due to unknown address (n = 84) or because patients felt too ill to answer the questionnaire (n = 99). The final participation rate in the survey was 64% (1432/2240).

### Study design and case definition

We conducted a case-control study, nested in the patient satisfaction survey. Cases were patients selected for the survey who did not send back a filled in questionnaire by the end of the data collection period (N = 808); controls were patients who did (N = 1432). Cases and controls were selected at random from the lists of non-respondents and respondents, using computer-generated random numbers. Medical and nursing records of cases and controls were retrieved together from departmental archives and abstracted by one of the authors (VK) who was blinded to case-control status. The case-report form had been pre-tested on 40 records before the main study by two authors (VK, LS) with a good one inter-rater agreement (Kappa coefficient > = 0.80 for all items).

### Independent variables

We produced a list of potential barriers based on previous studies of survey participation or of communication problems (see Introduction). We created a case-report form which included the following information: a) presence of a language barrier, defined by a difficulty to understand French and/or to communicate in French with the healthcare team by the search of a specific mention in the patient medical or nurse records and/or the use of interpreters during the hospital stay; and/or the mention “Does not speak French” on returned questionnaire; b) illiteracy, if mentioned in the patient medical or nurse records, c) presence of cognitive disorders defined by a positive minimal mental status or neuropsychological screening realized during the hospital stay, d) diagnosis of dementia or central nervous system disorder mentioned in the patient medical or nurse records, e) active alcohol and/or drug dependence, f) psychiatric disease described in the patient medical or nurse records or by the use of anti-depressives or psychotropic medications, g) sight deficiency, and h) difficulties in writing described in the patient medical or nurse records or by a positive scale on functional assessment. For each parameter, the answering items were categorical as “certainly yes”, “possibly” or “certainly no”. During analysis the answers were grouped as “present” if the answers were “certainly yes” or “possibly” were used and “absent” otherwise. Regarding language barriers, two variables were assessed: “difficulties in French understanding” coded as “no apparent difficulties”, “partial difficulties” or “does not understand French”, and “difficulties in French expression” coded as “no apparent difficulties”, “partial difficulties” and “does not speak French”. A positive language barrier was defined by the presence of difficulties in reading or speaking, complete or partial. The following socio-demographic variables were also retrieved: patient gender, birth date, nationality, dates of hospital admission and discharge, unit of hospitalization, destination after discharge (at home or other).

### Sample size estimation

At the time of the protocol writing and based on local data, we anticipated 10% of language difficulties among participants (controls). Taking an alpha error (two-sided) of 5% and a power of 90%, 2×200 patients would be necessary to show a difference in the proportion of language barrier of 12% between cases and controls, (22% versus 10%, which corresponds to an odds ratio of 2.5).

### Statistical analysis

Cases were compared to controls in using the Chi-square test or Fisher exact test (categorical variables) and Student t or Mann-Whitney tests (continuous variables). We examined the following associations with the outcome non-participation: language barrier, drug and alcohol abuse; cognitive impairment, psychiatric disorders and substance abuse. We constructed a parsimonious multiple logistic regression to identify independent determinants of non-participation. We used a forward stepwise procedure starting with the most significant variable associated with the outcome then introducing one by one the other variables that were significant in univariate analysis. All independent variables with a *P*<0.05 were kept in the final model. Goodness-of-fit of the model was assessed by the Hosmer-Lemeshow test. The amount of variation in the outcome explained by the model was indicated by the Pseudo R-square. The predictive validity of the multivariate model was assessed by the area under the receiver operating characteristic curve (AUC) and its 95% confidence interval (95% CI). We also used logistic regression to analyse the association between the number of significant predictors and non-participation. The association between the number of predictors and non-participation was also analysed. Finally the probability of non-participation was estimated by the presence of 0, 1, 2 or 3 risk factors after weighing the observed distributions by the inverse of sampling fractions of the cases and controls.

All analyses were performed using STATA IC 11 (STATA Corp., College Station, Texas, USA). Statistical significance was defined as *P*<0.05 (two-sided).

## Results

We included 197 cases and 205 controls. Among cases, stated reasons for non-participation were “Too ill to participate” (N = 20, 10.2%), “Does not speak French” (N = 13, 6.6%), “Does not want to participate” (N = 21, 10.7%). In addition, 8 patients sent back an empty questionnaire (4.1%) and 135 (68.5%) did not respond.

### Comparison of non-participants and participants

Cases and controls were similar in terms of age, gender and nationality (Table 1). Cases had longer hospital stays and were less likely to be discharged to their home compared to controls. A higher proportion of cases were hospitalized in Geriatrics and Psychiatry units compared to controls. Cases were more frequently illiterate than controls but this difference was not statistically significant. More cases than controls spoke a foreign language and more had minor or major difficulties in understanding French or communicating in French. Of note, 8 patients sent back the questionnaire with the mention “Does not speak French” but were not identified as having a language barrier in their medical chart. Altogether, the proportion of a language barrier was higher among cases (14.7% vs. 5.4%, *P* = 0.002) compared to controls.

**Table 1:** Patient characteristics among non-participants and participants in the 2005 patient satisfaction survey at the University Hospitals of Geneva.

Variables	Non-participants(cases, N = 197)	Participants(controls, N = 205)	p-value
Mean age in years (±SD)	53.1 (±20.9)	51.9 (±19.2)	0.56
Gender (women), n (%)	123 (62.4)	114 (55.6)	0.16
Nationality, n (%)			0.64
Swiss	112 (56.9)	126 (61.5)	
Other French-spoken countries	14 (7.1)	13 (6.3)	
Other	71 (36.0)	66 (32.2)	
Mean duration of hospital stay in days (±SD)	13.1 (±20.4)	8.9 (±17.1)	0.03^1^
Unit care of hospitalization, n (%)			<0.001
Internal medicine	33 (16.8)	45 (21.9)	
Geriatrics	14 (7.1)	1 (0.5)	
Surgery	48 (24.4)	76 (37.1)	
Psychiatry	41 (20.8)	13 (6.3)	
Neurosciences	25 (12.7)	25 (12.2)	
Gynecology-Obstetrics	36 (18.3)	45 (21.9)	
Patient outcome, n (%)			0.02
Discharged at home	165 (83.8)	188 (91.7)	
Other	32 (16.2)	17 (8.3)	
First spoken language, n (%)			0.06
French	104 (52.8)	127 (61.9)	
Other language	93 (47.2)	78 (38.1)	
Illiteracy, n (%)			0.49^2^
Yes	2 (1.0)	1 (0.5)	
Possible	4 (2.0)	2 (1.0)	
No	191 (97.0)	202 (98.5)	
French understanding, n (%)			0.003
Minor to major difficulties	23 (11.7)	8 (3.9)	
No difficulty	174 (88.3)	197 (96.1)	
Communication in French, n (%)			0.02
Minor to major difficulties	24 (12.2)	11 (5.4)	
No difficulty	173 (87.8)	194 (94.6)	
Interpreter services during hospital stay, n (%)			0.69
Yes	5 (2.5)	4 (2.0)	
No	192 (97.5)	201 (98.0)	
Cognitive limitations, n (%)			<0.001^2^
Yes	28 (14.2)	9 (4.4)	
Possible	4 (2.0)	0 (0)	
No	165 (83.8)	196 (95.6)	
Dementia/CNS deficiency, n (%)			0.006^2^
Yes	31 (15.7)	19 (9.3)	
Possible	5 (2.5)	0 (0)	
No	161 (81.7)	186 (90.7)	
Alcohol dependence, n (%)			<0.001
Yes	30 (15.2)	7 (3.4)	
Possible	8 (4.1)	6 (2.9)	
No	159 (80.7)	192 (93.7)	
Drug dependence, n (%)			0.001^2^
Yes	17 (8.6)	3 (1.5)	
Possible	2 (1.0)	1 (0.5)	
No	178 (90.4)	201 (98.1)	
Psychiatric history, n (%)			<0.001
Yes	63 (32.0)	38 (18.5)	
Possible	17 (8.6)	5 (2.4)	
No	117 (59.4)	162 (79.0)	
Sight deficiency, n (%)			0.06
Yes	12 (6.1)	8 (3.9)	
Possible	15 (7.6)	6 (2.9)	
No	170 (86.3)	191 (93.2)	
Communication deficiency (aphasia/autism), n (%)			0.17^2^
Yes	2 (1.0)	0 (0)	
Possible	1 (0.5)	4 (1.9)	
No	194 (98.5)	201 (98.1)	
Difficulties in writing, n (%)			0.77
Yes	7 (3.6)	10 (4.9)	
Possible	10 (5.1)	9 (4.4)	
No	180 (91.4)	186 (90.7)	

1 Mann-Whitney nonparametric test;

2 Fisher exact test.

Regarding medical determinants for non-participation, we also combined the “yes” and “possible” answers. More cases had a cognitive impairment (16.2% vs. 4.4%, *P*<0.001), dementia or a central nervous system deficiency (18.3% vs. 9.3%, *P* = 0.009), psychiatric history (40.6% vs. 21.0%, *P*<0.001), sight deficiency (13.7% vs. 6.8%, *P* = 0.06), or alcohol and/or drug dependence (24.9% vs. 7.8%, *P*<0.001) compared to controls. There was no difference between cases and controls in communication problems or difficulty in writing.

### Modelling non-participation

Because alcohol and drug dependence were correlated, we combined both addictions in one variable. The multivariate analysis identified five independent predictors for non-participation: language barrier, cognitive limitations, psychiatric diagnosis, alcohol and/or a drug dependence and sight deficiency (Table 2). Sight deficiency was forced into the model as it was near the limit of statistical significance (*P* = 0.06). The discriminative value of the multivariate logistic regression model was moderate with an area under the receiver operating characteristic curve of 0.72 (95%CI: 0.67–0.76).

**Table 2:** Independent predictors of non-participation in the 2005 satisfaction survey at the University Hospitals of Geneva.

	Odds Ratio	95% confidence interval	p-value
Language barrier	4.53	2.14–9.59	<0.001
Cognitive limitations	3.72	1.64–8.42	0.002
Sight deficiency	2.07	0.98–4.36	0.06
Drug or alcohol dependence	3.75	1.97–7.14	<0.001
Psychiatric diagnosis	1.99	1.23–3.23	0.005

The odds ratio for non-participation increased gradually with the number of predictors (between 0 and 3, none of the patients had 4 or 5 risk factors), as did the estimated proportion for non-participation calculated in our study population (Table 3).

**Table 3:** Association between the number of barriers and the non-participation in the patient survey (odds ratio and 95%CI) and the estimated probability of non-participation.

N (%)	Non-participants(N = 197)	Participants(N = 205)	Odds ratio(95% CI)	Estimated proportion of non-participants in original population
No barrier	56 (28.4)	133 (64.9)	1.0	19%
One barrier	79 (40.1)	52 (25.4)	3.6 (2.3–5.8)	46%
Two barriers	48 (24.4)	19 (9.3)	6.0 (3.2–11.1)	59%
Three barriers	14 (7.1)	1 (0.5)	33.2 (4.3–258.9)	89%

## Discussion

In this study, we have identified several barriers to participation in a patient satisfaction survey, i.e., factors that may interfere with the process of filling out a paper-based questionnaire. Difficulty in communicating in French, cognitive limitations, drug or alcohol dependence, psychiatric diagnosis, and sight problems were more frequent among non-participants than among participants. In contrast, illiteracy was uncommon in this sample and the difference between cases and controls, albeit in the expected direction, was not statistically significant. Similarly, aphasia and motor or neurologic alterations that interfere with writing were not associated with non-participation. Even if these predictors could be anticipated based on an *a priori* theory of non-response, only cognitive deficiency, and alcohol use has been linked with survey participation to date. This is the first study that documents the importance of a broad set of barriers in an actual patient survey.

The first important risk factor for non-participation was language. Canton Geneva has a mixed population, with 38.7% of foreigners of non French-spoken in 2005 [28], and the hospital admits a large proportion of patients who are not proficient in French. While this proportion was lower than expected, the difference between non-participants and participants was considerable. The second strong determinant of non-participation was substance abuse. The burden of alcohol abuse in Switzerland is high compared to other European countries [29]. Patients dependent on alcohol and/or drugs are more often dissocialized [30], depressed or anxious [31] and may have cognitive impairment [32], [33]; these are so many barriers to participation in a survey. Cognitive limitation was another independent risk factor for non-participation. Jacomb et al. [21] showed in a longitudinal survey of elderly patients that those with a cognitive impairment were less willing to participate. Cognitive impairment may lead a patient to misunderstand the study documents, including its purpose and what participation entails, or even the meaning of questions that are asked [34], [35]. This may lead to the refusal to participate [22]. Finally the presence of mental illness was also associated with non-participation. This confirms previous observations of low participation rates in surveys of psychiatric patients [3], [26]. The mechanisms of this phenomenon are likely complex. E.g., schizophrenia [36], [37] or thought disorders [27] have been associated with neurocognitive deficits that may influence decisional capacity including understanding, appreciation, reasoning and decision making, which may affect participation in surveys.

This study has strengths and limitations. We are not aware of other studies assessing the role of language barriers, substance abuse, or sight deficiency in the inability to participate in a patient survey, so this study fills a gap in knowledge. We restricted the risk of information bias by blinding the research assistant responsible for data extraction to case-control status. The quality of data extraction was pre-tested between two authors in order to assure a Kappa coefficient above 0.80. The main limitation of this study is that information was abstracted from medical and nursing charts, where information is not always recorded with a high level of accuracy. Consequently it is likely that most barriers we examined were underreported, particularly information on language barriers or illiteracy. However, this likely resulted in non-differential misclassification bias, which would weaken the true associations. For instance, for 8 cases, no language barrier was identified in the medical charts, yet the patient sent back a questionnaire with the mention “Does not speak French”. Similarly, the prevalence of illiteracy was lower in our sample than would be expected from other sources [38]. While the use of interpreters may also appear to be underestimated – only 2.2% of the study population used interpreters while 8.7% reported a language barrier – independent evidence suggests that interpreters are underused at this hospital [39]. Another limitation is the debatable generalizability of our results. As Geneva is particularly multicultural, the importance of language barriers may be greater than in other settings; similarly, the importance of cognitive limitations or of alcohol and drug use would be lower in population-based surveys. Finally we did not demonstrate that the uneven likelihood of participation led to bias in the variable of interest, i.e., patient satisfaction. . In a previous study [26], we have shown that the bias caused by non-participation was moderate in the whole survey; however, this does not necessarily rule out stronger bias in specific patient subgroups. Other previous studies suggest that selection based on cognitive impairment or the demographic characteristics do not necessarily lead to bias [9], [10].

These results raise the question of what should be done to facilitate the participation of patients who have one or more risk factors for not completing a survey. This will depend on the nature of the barrier. Language difficulties can be removed by the use of interpreters [39], or in some instances by the translation of the survey documents [40]. Some sensory or motor limitations will be bypassed by the use of in-person interviewing. For cognitive limitations, mental illness or substance abuse, proxy respondents may be considered. Proxy respondents usually provide reliable factual information [41] and cause limited biases [42], [43], but whether this holds also for subjective assessments such as satisfaction with health care requires further study. Health surveys do require a level of cognitive ability and motivation that may be out of reach for a substantial proportion of the target population. However strategies to improve participation can be implemented only if potential barriers are identified.

In conclusion, this study has identified five barriers to non-participation in mail surveys that aim to measure patient satisfaction or other patient outcomes that are relevant for quality assessment. Patients who suffer from these impediments may be underrepresented in quality assessment, and their experiences may not be taken into account in quality improvement. Alternative survey strategies are needed.

## References

[1] Rosenthal GE, Shannon SE (1997). The use of patient perceptions in the evaluation of health-care delivery systems.. Med Care.

[2] Young GJ, Meterko M, Desai KR (2000). Patient satisfaction with hospital care: effects of demographic and institutional characteristics.. Med Care.

[3] Peytremann-Bridevaux I, Scherer F, Peer L, Cathieni F, Bonsack C (2006). Satisfaction of patients hospitalised in psychiatric hospitals: a randomised comparison of two psychiatric-specific and one generic satisfaction questionnaires.. BMC Health Serv Res.

[4] Hartge P (1999). Raising response rates: getting to yes.. Epidemiology.

[5] Mazor KM, Clauser BE, Field T, Yood RA, Gurwitz JH (2002). A demonstration of the impact of response bias on the results of patient satisfaction surveys.. Health Serv Res.

[6] Etter JF, Perneger TV (1997). Analysis of non-response bias in a mailed health survey.. J Clin Epidemiol.

[7] Ross S, Grant A, Counsell C, Gillespie W, Russell I (1999). Barriers to participation in randomised controlled trials: a systematic review.. J Clin Epidemiol.

[8] Boshuizen HC, Viet AL, Picavet HS, Botterweck A, van Loon AJ (2006). Non-response in a survey of cardiovascular risk factors in the Dutch population: determinants and resulting biases.. Public Health.

[9] Kjoller M, Thoning H (2005). Characteristics of non-response in the Danish Health Interview Surveys, 1987–1994.. Eur J Public Health.

[10] Holt VL, Martin DP, LoGerfo JP (1997). Correlates and effect of non-response in a postpartum survey of obstetrical care quality.. J Clin Epidemiol.

[11] Asch DA, Christakis NA (1994). Different response rates in a trial of two envelop styles in mail survey research.. Epidemiology.

[12] Groves R, Fowler F, Couper M, Lepkowski J, Singer E, Groves R, Fowler F, Couper M, Lepkowski J, Singer E, Tourangeau R (2004). Nonresponse in sample surveys.. Survey methodology.

[13] Penckofer S, Byrn M, Mumby P, Ferrans CE (2011). Improving subject recruitment, retention, and participation in research through Peplau's theory of interpersonal relations.. Nurs Sci Q.

[14] Edwards P, Roberts I, Clarke M, DiGuiseppi C, Pratap S (2007). Methods to increase response rates to postal questionnaires.. Cochrane Database Syst Rev.

[15] Nakash RA, Hutton JL, Jorstad-Stein EC, Gates S, Lamb SE (2006). Maximising response to postal questionnaires–a systematic review of randomised trials in health research.. BMC Med Res Methodol.

[16] Nelson KM, Geiger AM, Mangione CM (2004). Racial and ethnic variation in response to mailed and telephone surveys among women in a managed care population.. Ethn Dis.

[17] Rendell JM, Merritt RD, Geddes JR (2007). Incentives and disincentives to participation by clinicians in randomised controlled trials.. Cochrane Database Syst Rev.

[18] Harmsen JA, Bernsen RM, Bruijnzeels MA, Meeuwesen L (2008). Patients' evaluation of quality of care in general practice: what are the cultural and linguistic barriers?. Patient Educ Couns.

[19] Schouten BC, Meeuwesen L, Tromp F, Harmsen HA (2007). Cultural diversity in patient participation: the influence of patients' characteristics and doctors' communicative behaviour.. Patient Educ Couns.

[20] Pignone M, DeWalt DA, Sheridan S, Berkman N, Lohr KN (2005). Interventions to improve health outcomes for patients with low literacy. A systematic review.. J Gen Intern Med.

[21] Jacomb PA, Jorm AF, Korten AE, Christensen H, Henderson AS (2002). Predictors of refusal to participate: a longitudinal health survey of the elderly in Australia.. BMC Public Health.

[22] Hebert R, Bravo G, Korner-Bitensky N, Voyer L (1996). Refusal and information bias associated with postal questionnaires and face-to-face interviews in very elderly subjects.. J Clin Epidemiol.

[23] Wild TC, Cunningham J, Adlaf E (2001). Nonresponse in a follow-up to a representative telephone survey of adult drinkers.. J Stud Alcohol.

[24] Tolonen H, Laatikainen T, Helakorpi S, Talala K, Martelin T (2010). Marital status, educational level and household income explain part of the excess mortality of survey non-respondents.. Eur J Epidemiol.

[25] Wall M, Teeland L (2004). Non-participants in a preventive health examination for cardiovascular disease: characteristics, reasons for non-participation, and willingness to participate in the future.. Scand J Prim Health Care.

[26] Perneger TV, Chamot E, Bovier PA (2005). Nonresponse bias in a survey of patient perceptions of hospital care.. Med Care.

[27] Candilis PJ, Geppert CM, Fletcher KE, Lidz CW, Appelbaum PS (2006). Willingness of subjects with thought disorder to participate in research.. Schizophr Bull.

[28] Frei D (2006). Statistical memento of Canton Geneva, 2006.

[29] Rehm J, Taylor B, Roerecke M, Patra J (2007). Alcohol consumption and alcohol-attributable burden of disease in Switzerland, 2002.. Int J Public Health.

[30] Gmel G, Rehm J (2003). Harmful alcohol use.. Alcohol Res Health.

[31] Conner KR, Pinquart M, Gamble SA (2009). Meta-analysis of depression and substance use among individuals with alcohol use disorders.. J Subst Abuse Treat.

[32] Sullivan EV, Pfefferbaum A (2005). Neurocircuitry in alcoholism: a substrate of disruption and repair.. Psychopharmacology (Berl).

[33] Green A, Garrick T, Sheedy D, Blake H, Shores EA (2010). The effect of moderate to heavy alcohol consumption on neuropsychological performance as measured by the repeatable battery for the assessment of neuropsychological status.. Alcohol Clin Exp Res.

[34] Okonkwo OC, Griffith HR, Copeland JN, Belue K, Lanza S (2008). Medical decision-making capacity in mild cognitive impairment: a 3-year longitudinal study.. Neurology.

[35] Jefferson AL, Lambe S, Moser DJ, Byerly LK, Ozonoff A (2008). Decisional capacity for research participation in individuals with mild cognitive impairment.. J Am Geriatr Soc.

[36] Jeste DV, Depp CA, Palmer BW (2006). Magnitude of impairment in decisional capacity in people with schizophrenia compared to normal subjects: an overview.. Schizophr Bull.

[37] Palmer BW, Dunn LB, Depp CA, Eyler LT, Jeste DV (2007). Decisional capacity to consent to research among patients with bipolar disorder: comparison with schizophrenia patients and healthy subjects.. J Clin Psychiatry.

[38] Williams MV, Baker DW, Parker RM, Nurss JR (1998). Relationship of functional health literacy to patients' knowledge of their chronic disease. A study of patients with hypertension and diabetes.. Arch Intern Med.

[39] Hudelson P, Vilpert S (2009). Overcoming language barriers with foreign-language speaking patients: a survey to investigate intra-hospital variation in attitudes and practices.. BMC Health Serv Res.

[40] Bischoff A, Tonnerre C, Eytan A, Bernstein M, Loutan L (1999). Addressing language barriers to health care, a survey of medical services in Switzerland.. Soz Praventivmed.

[41] Cusick CP, Gerhart KA, Mellick DC (2000). Participant-proxy reliability in traumatic brain injury outcome research.. J Head Trauma Rehabil.

[42] Duncan PW, Lai SM, Tyler D, Perera S, Reker DM (2002). Evaluation of proxy responses to the Stroke Impact Scale.. Stroke.

[43] Groves R, Fowler F, Couper M, Lepkowski J, Singer E, Groves R, Fowler F, Couper M, Lepkowski J, Singer E, Tourangeau R (2004). Questions and answers in surveys.. Survey Methodology.

